# An Interactive, Asynchronous Intimate Partner Violence Module for Medical Students: Improving Preparedness, Confidence, and Knowledge

**DOI:** 10.15766/mep_2374-8265.11618

**Published:** 2026-07-14

**Authors:** Victoria Ivy Petsinger, Sugapradha Saravanan, Rahul Manne, Ramzi W. Nahhas, Bethany Harper, Larrilyn Grant

**Affiliations:** 1 Medical Student, Wright State University Boonshoft School of Medicine; 2 Associate Professor, Wright State University Boonshoft School of Medicine Department of Population and Public Health Sciences, Department of Psychiatry; 3 Associate Professor, Wright State University Boonshoft School of Medicine Department of Psychiatry; 4 Assistant Professor, Wright State University Boonshoft School of Medicine Department of Psychiatry; † Co-primary author

**Keywords:** Intimate Partner Violence, Trauma-Informed Care, Asynchronous Learning, Psychiatry

## Abstract

**Introduction:**

Intimate partner violence (IPV) is a cyclical pattern of behavior that can cause physical, psychological, and/or sexual harm with significant risks, including posttraumatic stress disorder. The primary aim of IPV is to establish or maintain power over the partner. While many organizations recommend screening for IPV, there is limited exposure to IPV in medical school curricula. This critical gap represents opportunity for further education. Thus, an interactive module on IPV was created for medical students on their psychiatry clerkship to teach skills in assessment, treatment planning, and resources. This study evaluates the module's effectiveness and medical students’ readiness in addressing IPV in clinical settings.

**Methods:**

The module provides a structured overview of IPV, including epidemiology, barriers to seeking help, and screening approaches. It incorporates video-based clinical scenarios, allowing learners to observe and practice how to recognize and respond to IPV in real-world settings. Pre- and postmodule surveys were administered to assess changes in knowledge, confidence, and preparedness in addressing IPV. Individual item scores were summed within each domain, and pre- and postmodule total scores were compared using paired *t* tests.

**Results:**

Data were collected from 122 third-year medical students. Students showed statistically significant improvements in knowledge and reported higher confidence and preparedness (*P* < .001) after completing the module. More students correctly identified risk factors, felt adequately trained, and were more comfortable discussing IPV.

**Discussion:**

The IPV module improved students’ knowledge, confidence, and preparedness in addressing IPV. Future studies should assess long-term retention and its impact on clinical practice.

## Educational Objectives

By the end of the activity, learners will be able to:

1. Demonstrate increased knowledge of the health complications and epidemiologic factors associated with intimate partner violence.
2. Report increased comfort with screening for and discussing intimate partner violence in clinical settings.
3. Report increased preparedness to recognize and respond to patients experiencing intimate partner violence.

## Introduction

Intimate partner violence (IPV) remains a pervasive public health crisis in the United States (US), affecting individuals across all demographics.^1^ According to the US Centers for Disease Control and Prevention^1^ (CDC), approximately 1 in 4 women and 1 in 10 men report experiencing IPV in their lifetime, which may include physical, sexual, psychological, or economic abuse. IPV impacts one's emotional, psychological, physical, and financial well-being, while also placing a substantial burden on health care systems and educational institutions responsible for training future professionals. According to Peterson et al.,^2^ the estimated IPV lifetime cost among approximately 43 million US adults with a history of experiencing IPV was nearly $3.6 trillion.

Survivors of IPV frequently endure depression, anxiety, posttraumatic stress disorder (PTSD), and suicidal ideation.^3^ A systematic review and meta-analysis identified strong associations between psychological violence and mental health disorders, with particularly large effect sizes for depression and PTSD among women.^4^ These effects often persist long after the abusive relationship ends, affecting survivors’ ability to function in daily life and maintain relationships.^4^

IPV survivors may also suffer acute injuries such as bruises, fractures, and head trauma, as well as chronic conditions including gastrointestinal disorders, migraines, and chronic pain.^5^ IPV during pregnancy is associated with heightened risk of miscarriage, preterm labor, and fetal injury, often resulting in long-term medical complications and increased health care utilization.^6^

Economic abuse, a frequently overlooked aspect of IPV, can be equally debilitating. Abusers may restrict survivors’ access to financial resources, sabotage employment opportunities, or accumulate debt in the victim's name, leading to prolonged economic dependence. Additionally, IPV contributes to substantial societal costs through lost productivity and increased health care expenditures, with the CDC estimating the economic burden in the billions each year.^7^

Despite the prevalence and impact, IPV remains insufficiently addressed in medical, nursing, and graduate health education. Studies show that even after formal training, health care professionals often feel ill-prepared to identify and respond to IPV.^8^ Medical students similarly report limited training and low confidence in managing IPV cases. A 2019 study investigated 204 health care providers, 27 of which were social and behavioral health workers. These health care professionals specifically required more preparation, knowledge, and survivor understanding regarding IPV training. Even with the extra training, these 27 reported the lowest screening rates of IPV compared to other health care personnel groups.^9^ This lack of confidence was also apparent in a study that adapted the Physician Readiness to Manage Intimate Partner Violence Survey (PREMIS) for pharmacy students. A total of 144 surveys were collected, and the results showed low levels of knowledge in recognizing and treating IPV.^10^ These educational deficiencies hinder health care professionals’ ability to identify and support IPV survivors effectively.

Most existing IPV curricula for medical students are short term (less than 1 academic year) and rely primarily on formal lectures and standardized patients as delivery methods.^8^ Standardized patient interventions have shown to be effective, with 1 study demonstrating significant improvements in comfort recognizing IPV signs and knowledge of resources.^9^ However, these approaches require substantial resources and faculty time, which are commonly cited as the biggest implementation barriers.^9^ Medical schools in Australia report a median time of only 3–6 contact hours for IPV education, with time constraints and resource shortages as key barriers.^10^ Existing curricula also demonstrate significant gaps in addressing treatment planning and resource navigation. A scoping review found that most curricula taught risk factors and identification of IPV but lacked comprehensive coverage of management and intervention strategies.^8^ This is problematic because screening alone without intervention does not improve outcomes. Rather, effective approaches require ongoing support services, counseling, and connection to community resources. Additionally, published curricula show great variation in assessment methods without consistent validated tools for measuring effectiveness, making it difficult to establish competency standards.^8^

This module is novel in several key areas. It represents an asynchronous, interactive module format that has also been updated to meet federal accessibility standards, ensuring usability for a broad range of learners. While most published curricula use formal lectures (resource-intensive) or standardized patients (expensive and time-limited), asynchronous e-learning modules offer scalability and flexibility that address the primary implementation barrier of limited time in medical education.

The use of an interactive asynchronous module aligns with several educational principles. Interactive modules allow students to engage actively with content rather than passively receiving information. Evidence shows that interventions based on role-plays and feedback are more effective than lectures alone for communication skills development. Video-recorded peer role-play with standardized checklists has demonstrated effectiveness in developing communication competencies among first-year medical students, with 87% agreeing that it was an effective learning method.^10^ Asynchronous formats support learner autonomy and self-paced engagement, which is particularly important for sensitive topics where students may need time for reflection and emotional processing. Medical students report that trauma-informed teaching strategies, including preparedness for teaching and appropriate debriefing, promote psychological safety when learning about sensitive topics like domestic violence.^11^ While some evidence suggests synchronous learning may reduce cognitive load compared to asynchronous formats, asynchronous modules allow students to pause, reflect, and revisit complex content, which is particularly valuable for learning communication skills around sensitive topics that may trigger emotional responses.

The purpose of this module is to provide medical students with a structured framework for understanding IPV. The module highlights key risk factors that contribute to IPV, outlines its harmful effects on survivors, and describes clinical signs and symptoms that may indicate abuse. It also guides learners through evidence-based steps for supporting survivors and connecting them to appropriate resources and care. This module is oriented to the baseline material of dealing with common presentations of IPV and does not address the more complex, nuanced presentations.

Currently, there are several free training resources available that are appropriate for third-year medical students learning about IPV. The World Health Organization (WHO) offers a curriculum titled “Caring for Women Subjected to Violence: A WHO Curriculum for Training Health Care Providers.”^11^ This comprehensive curriculum is specifically designed for health care provider training and includes structured modules that can be adapted for self-directed learning. The Futures Without Violence Association offers free educational videos on the CUES approach for IPV (Confidentiality, Universal Education and Empowerment, Support).^12^ The Agency for Healthcare Research and Quality also offers a comprehensive table of IPV screening instruments with items, scoring, and citations, which can serve as self-study resources for learning validated assessment tools.^13^ Despite the availability of these resources, there remains a gap for interactive, case-based IPV training in medical education. Our asynchronous module addresses this through clinical decision-making and patient-centered communication.

## Methods

We developed an asynchronous, self-paced, online module as part of the third-year psychiatry clerkship at a midwestern medical school. Our team, comprising 2 psychiatrists specializing in medical education and a group of psychiatry residents and medical students interested in medical education, used Articulate software to create the course. Literature supports the use of realistic case-based scenarios to promote critical thinking, problem analysis, and reflective decision-making in online learning environments. To that end, we adapted a clinical case originally used in a live session on IPV and modified it for self-guided digital learning.^11,14,15^ The module takes approximately 1 hour to complete.

The curriculum is centered around a longitudinal case of a 21-year-old patient experiencing IPV across outpatient, emergency, inpatient psychiatry, and follow-up settings. Through this case, learners engage with core concepts such as IPV definitions, trauma-informed interviewing, clinical and legal considerations, barriers to care, case conceptualization, and safety planning.

The module incorporates interactive components to reinforce learning, including branched patient interview scenarios, multiple choice questions, and sorting/matching exercises for topics such as identifying risk factors, warning signs, and stages of abuse. Videos throughout the module portray patient–provider interactions that highlight nonverbal cues and clinical decision-making. Additional activities include differential diagnosis exercises, reflective prompts (eg, countertransference), and exposure to community resources, including an audio recording of a hotline phone call with an advocate. Embedded expert commentary is provided throughout to emphasize key teaching points. Appendix A contains the full course. This course has been updated to meet federal accessibility standards.

All third-year medical students in the 2024–2025 academic year completed the course during the fourth week of their psychiatry clerkship. Students had no prior required IPV-specific training, but they had completed standard clerkship orientation and initial inpatient exposure by that time. No special audiovisual equipment or in-person facilitation was needed for implementation.

We assessed learner outcomes using pre- and postmodule surveys (Appendix B) adapted from the validated PREMIS.^16^ Terminology throughout the manuscript was revised, when possible, to reflect trauma-informed language. Terms such as “victim” and “batterer” were kept only when referencing the validated PREMIS instrument and corresponding survey items. We obtained informed consent to use de-identified survey data for research purposes and provided students with information about Family Educational Records Protection Act (FERPA) protections and the voluntary nature of participation (see Appendix B).

We administered the survey electronically before and after module completion. Each Likert-scale item was converted to a numerical value, beginning at 0. We reverse-coded negatively worded items so that higher scores reflected greater agreement in the intended direction. One knowledge item was excluded due to poor contribution to internal consistency. Item-level analysis demonstrated that removing a question related to appropriate IPV screening practices increased Cronbach's alpha from 0.49 at each time (pre and post) to 0.568 (pre) and 0.583 (post), and it was therefore excluded from the total knowledge score. We calculated total scores for preparation (8 items, 0–6 scale), knowledge (26 items, scored 1 if correct), and agreement (15 items, 0–4 scale). Total scores were then rescaled so that each was in the range 0 to 100. Cronbach's alpha indicated strong internal consistency for preparation (α = 0.919 pre, 0.951 post), moderate for agreement (α = 0.692 pre, 0.761 post), and lower for knowledge (α = 0.568 pre, 0.583 post).

We computed descriptive statistics (means, standard deviations, frequencies, proportions) for each item and total scores at both time points.^17^ Individual ordinal items were compared between times using a cumulative link mixed model (CLMM); individual binary items were compared using McNemar's test; and each total was compared between times using a paired-samples *t* test.

We received IRB approval for this study from Wright State University (IRB-2023-411), and the study was determined to be exempt.

We conducted data processing and analysis in R version 4.3.2.^18^ We used the ordinal package to fit CLMMs.^19^ All tests were 2-tailed, with α set at 0.05.

## Results

A total of 122 out of 131 third-year medical students (93.1%) consented to participate in the study and completed both the pre- and postmodule surveys. Most participants were between 25 and 29 years old, with a majority identifying as female.

### Knowledge Outcomes

Student knowledge showed a small but statistically significant increase following the module (mean difference = 2.77, 95% CI = 1.45, 4.10, *P* < .001). Correct identification of “gender female” as the strongest IPV risk factor increased from 27.9% to 54.9% (*P* < .001), and recognition that batterers use violence to control their partners increased from 83.6% to 94.3% (*P* < .001). Other items demonstrated little change, including recognition of abuse signs (from 82.0% to 82.8%, *P* = 1.000) and reasons survivors may not leave (from 91.0% to 91.8%, *P* = 1.000). For the multiselect item assessing general knowledge of IPV, the proportion of students selecting all correct options increased from 66.4% to 73.8% (*P* = .110). Full results are summarized in Table 1.

**Table 1.**
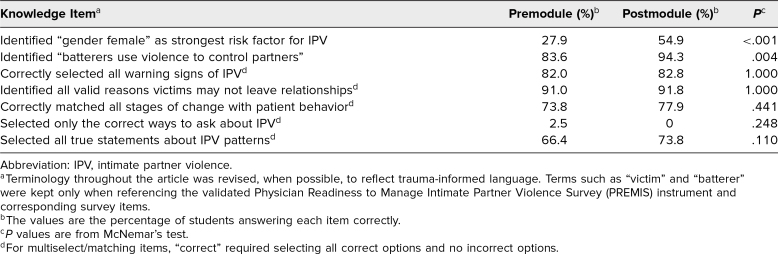
Pre- and Postmodule Knowledge Item Performance (*N* = 122)

### Perceptions and Confidence

Perceptions and confidence improved significantly following the module (mean difference in total score = 12.04, 95% CI = 10.32, 13.75, *P* < .001), reflecting greater comfort and more positive attitudes toward identifying and responding to IPV.

Students demonstrated important shifts in perceptions: strong disagreement with “If an IPV victim does not acknowledge the abuse, there is very little that I can do to help” increased from 20.5% to 35.2% (*P* = .002). Confidence in screening also improved—those strongly agreeing with “I will ask all new patients about abuse in their relationships” nearly doubled, increasing from 24.6% to 48.4% (*P* < .001). Perceived training gaps decreased, with the proportion of students disagreeing that they lacked sufficient training rising from 27.0% to 74.6% (*P* < .001).

Comfort discussing IPV also grew markedly. Agreement with the statement “I feel comfortable discussing IPV with my patients” increased from 32.0% to 84.4% (*P* < 0.001). Disagreement that students lacked skills to discuss abuse with a female, male, or gender-diverse individual rose substantially (female 43.4% to 82.0%, male 37.7% to 77.0%, and gender diverse 37.7% to 74.6%; *P* < .001 each).

Recognition of provider responsibility also increased. Strong agreement that “Health care providers have a responsibility to ask all patients about IPV” rose from 39.3% to 59.8% (*P* < .001), whereas agreement that providers lack the time or knowledge to help decreased significantly (4.9% to 0%, *P* = .018; 6.6% to 2.4%, *P* = .005; respectively).

Empathy and validation improved as well, with greater agreement that survivors may have valid reasons for remaining in abusive relationships (68.1% to 77.9%, *P* = .050). Students also became less concerned that screening would offend patients as strong disagreement with this concern increased (11.5% to 22.1%), although this change was not statistically significant (*P* = .149).

Examples of 5 items in this category with agreement levels pre- and postmodule and *P* values are provided in Table 2.

**Table 2.**
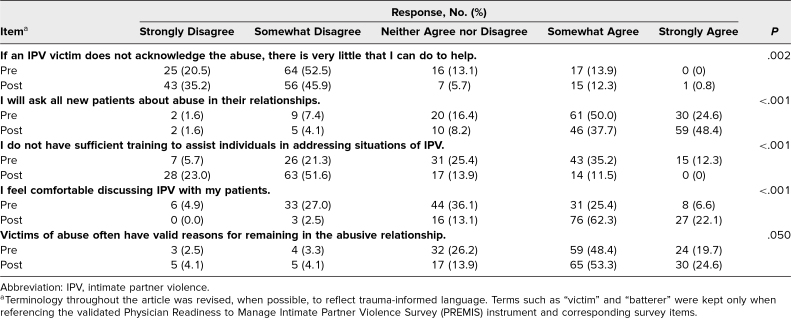
Pre- and Postmodule Perceptions and Confidence Item Performance (*N* = 122)

### Preparedness

Students reported a large and statistically significant increase in preparedness to address IPV following the module (mean difference = 29.27, 95% CI = 26.17, 32.36, *P* < .001). As shown in the Figure, almost every student increased their total score postmodule. Improvements were observed across all domains, including identifying risk factors and signs of IPV, responding to disclosures, understanding IPV in pregnancy, providing referrals, developing safety plans, and documenting IPV in the medical record. Collectively, these findings indicate broad gains in students’ confidence and skills in managing IPV in clinical settings. Examples of 5 items in this category with preparedness levels pre- and postmodule and *P* values are summarized in Table 3.

**Figure.**
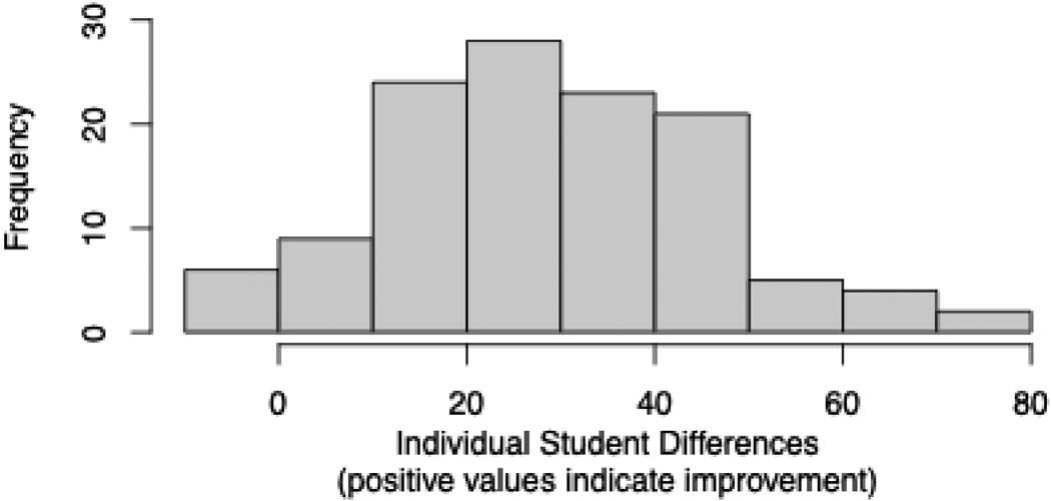
Individual student change in total preparedness score (postmodule score – premodule score, each rescaled to 0–100; positive values indicate improvement) (mean = 29.3, *SD* = 17.3, 95% CI = 26.2, 32.4).

**Table 3.**
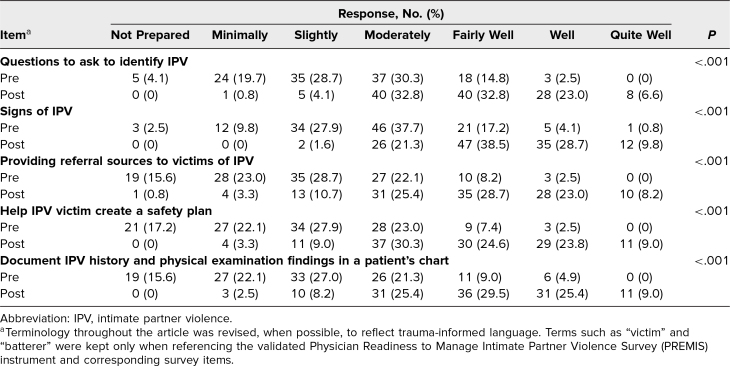
Pre- and Postmodule Preparedness Item Performance (*N* = 122)

## Discussion

This study demonstrates that our IPV learning module produced statistically significant improvements in knowledge, perceptions and confidence, and preparedness among third-year medical students. The largest gains were observed in preparedness, with more modest improvements in perceptions and confidence, and the smallest change in knowledge. These findings suggest that while students may enter clinical training with baseline knowledge about IPV, structured educational approaches can meaningfully enhance their readiness to apply IPV-related skills in clinical practice.

These results align with prior literature demonstrating that IPV education improves learner knowledge, attitudes, and readiness, while also highlighting ongoing challenges in translating knowledge into clinical practice.^8^ Given the limited coverage of IPV training in medical education, this asynchronous module addresses a critical educational need by combining conceptual knowledge and practical clinical strategies in a scalable, flexible, and accessible format for resource-limited curricula.

### Knowledge

The module improved students’ factual understanding of IPV, including recognition of epidemiologic risk factors (eg, female gender), patterns of abusive behavior and injury patterns. However, some items showed little change due to high baseline scores, whereas others revealed gaps in instruction. For example, performance on a multiselect item assessing appropriate ways to ask about IPV was very poor both before and after the module, suggesting the need for clearer teaching or question revision. These mixed results highlight both the strengths of the module in reinforcing foundational knowledge and the need for more explicit instruction around nuanced clinical communication skills, such as trauma-informed questioning, through interactive or clinically integrated approaches.

### Perceptions and Confidence

Students reported increased confidence in screening for and discussing IPV, with marked improvements in their willingness to ask about abuse and their comfort interacting with survivors across gender identities. These findings suggest that the module helped normalize IPV as a clinical topic and reinforced provider responsibility. At the same time, perceptions of systemic barriers (eg, limited provider time) shifted only modestly, underscoring structural challenges that extend beyond individual training. While self-reported confidence increased, this module represents an introductory educational experience, and we cannot conclude that these gains translate to clinical competence. Without supervised clinical encounters, students may overestimate their readiness to manage complexities of IPV care, including safety considerations, longitudinal care, and family dynamics.

### Preparedness

Preparedness showed the greatest improvement, particularly in safety planning, referrals, and documenting IPV in the medical record. These findings suggest that interactive, case-based learning may be effective in supporting the development of actionable IPV-related clinical skills. However, preparedness was assessed through self-report rather than observation. Future curricula should incorporate supervised clinical practices, such as standardized patient encounters, to evaluate competency directly.

### Implications

These findings have important implications for medical education. While brief educational modules can improve student knowledge, perceptions, confidence, and preparedness, they should be framed as foundational experiences within a broader, longitudinal curriculum. Integrating IPV training across multiple clerkships and incorporating supervised clinical encounters can support retention and promote clinical application. Additionally, addressing perceived systemic barriers, such as time constraints, may require institutional and curricular support within clinical environments.

### Limitations

This study has several limitations. First, knowledge retention was not assessed longitudinally; long-term impact remains unknown. Second, outcomes were based on self-report, which may not reflect true clinical performance. Finally, results from a single cohort at 1 institution limit generalizability.

### Future Directions

Future research should include multiple institutions and cohorts, with longitudinal assessments to evaluate knowledge retention and skill application over time. Expanding IPV training into preclerkship curricula and reinforcing this during clerkships could ensure progressive skill development. Preclinical training can emphasize IPV identification, epidemiology, and case-based discussions. Extending this training beyond psychiatry to other clerkships would promote interprofessional engagement and strengthen the health care system's ability to respond to IPV comprehensively. The module can be tailored for specialty-focused scenarios that would involve IPV. Incorporating standardized patient encounters and observed history-taking exercises would provide opportunities to assess competency directly.

Future iterations of the module could incorporate additional scenarios involving different cultural and language considerations (eg, addressing IPV in non–English-speaking patients and incorporating different cultural barriers to IPV so that a larger number of patients can be offered resources), overt aggression, substance involvement, and child protection considerations to further reflect the complexity of IPV in clinical practice. Future research can focus on the impact of incorporating such changes and assessing these skills at various points in time to determine the long-term impact of the IPV training and retention of skills.

## Appendices


IPV Articulate Module FolderIPV Pre- and Postmodule Survey.docx
*All appendices are peer reviewed as integral parts of the Original Publication.*
