# Supplementary material for: An Interactive, Asynchronous Intimate Partner Violence Module for Medical Students: Improving Preparedness, Confidence, and Knowledge
Source: MedEdPORTAL. 2026 Jul 14;22:11618. doi: 10.15766/mep_2374-8265.11618 (PMC13364887; doi:10.15766/mep_2374-8265.11618)
Supplement: Supplementary file 1 — IPV Articulate Module FolderIPV Pre- and Postmodule Survey.docx [file mep_2374-8265.11618-s001.zip › A. IPV Articulate Module Folder/FILE ACCESS INSTRUCTIONS.docx]

# Instructions for Accessing Interactive Module *MedEdPORTAL* Appendices

To facilitate the access and review of offline module (i.e., Articulate 360) appendices, which are submitted as .zip files and need to be unzipped to view the module. For a PC you need to download, extract, and launch. For a MAC you need to download and launch. See details and screenshots below.

**DOWNLOAD** (PC or MAC)

1. Download the module .zip file appendix to your desktop.
2. In your Download folder, locate the .zip file (MAC users skip to step 4).

**EXTRACT** (PC only)

1. Extract the file either by:
   1. Right clicking on the .zip file, selecting Extract all, and clicking Extract when prompted:

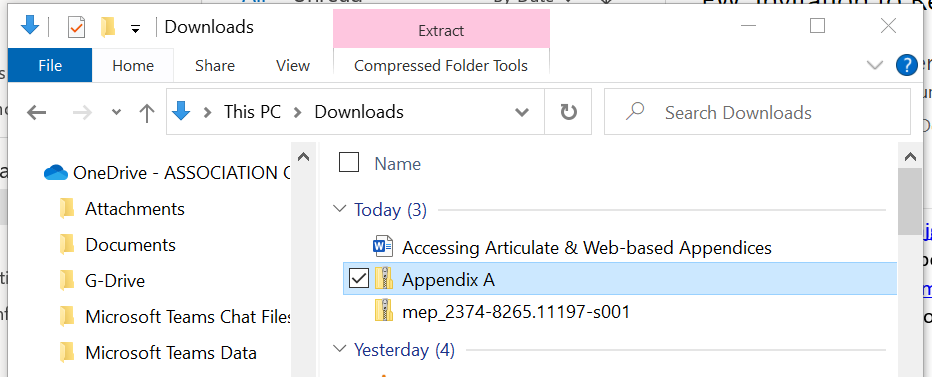

   2. Opening the .zip file, selecting Extract all from toolbar, and clicking Extract when prompted:


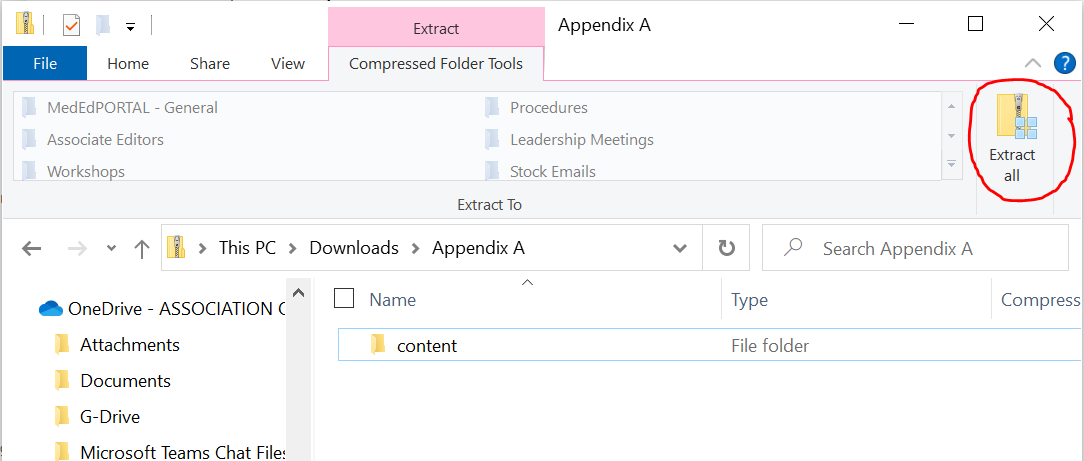


**LAUNCH** (PC or MAC)

1. An unzipped version of the folder should now appear on your desktop.


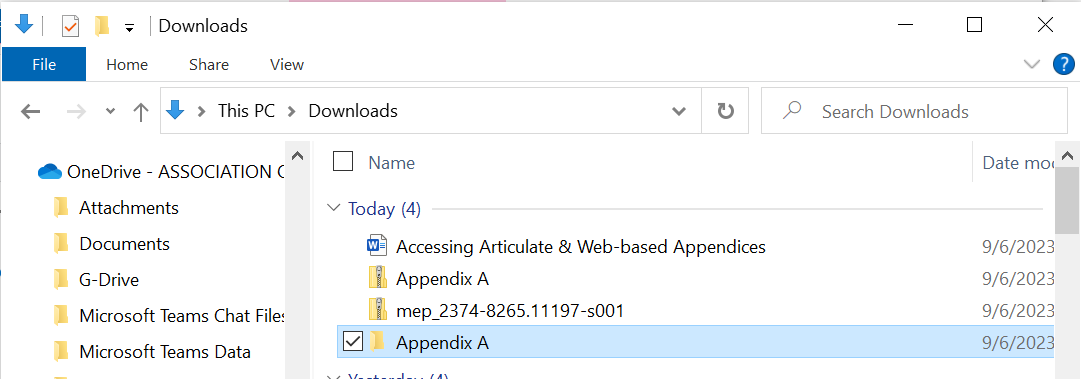


1. Open this unzipped appendix folder and click on the module subfolder (sometimes named content).

1. Double click on the index or story file (this .html file has a web-browser logo and may have other names).
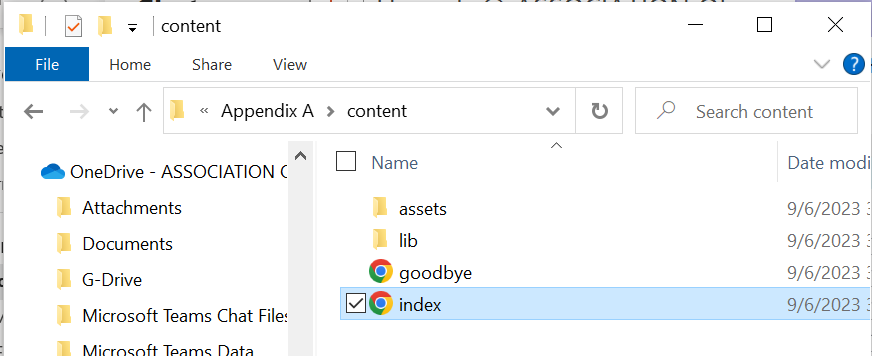

2. A web browser will open containing the navigable presentation for you to review.


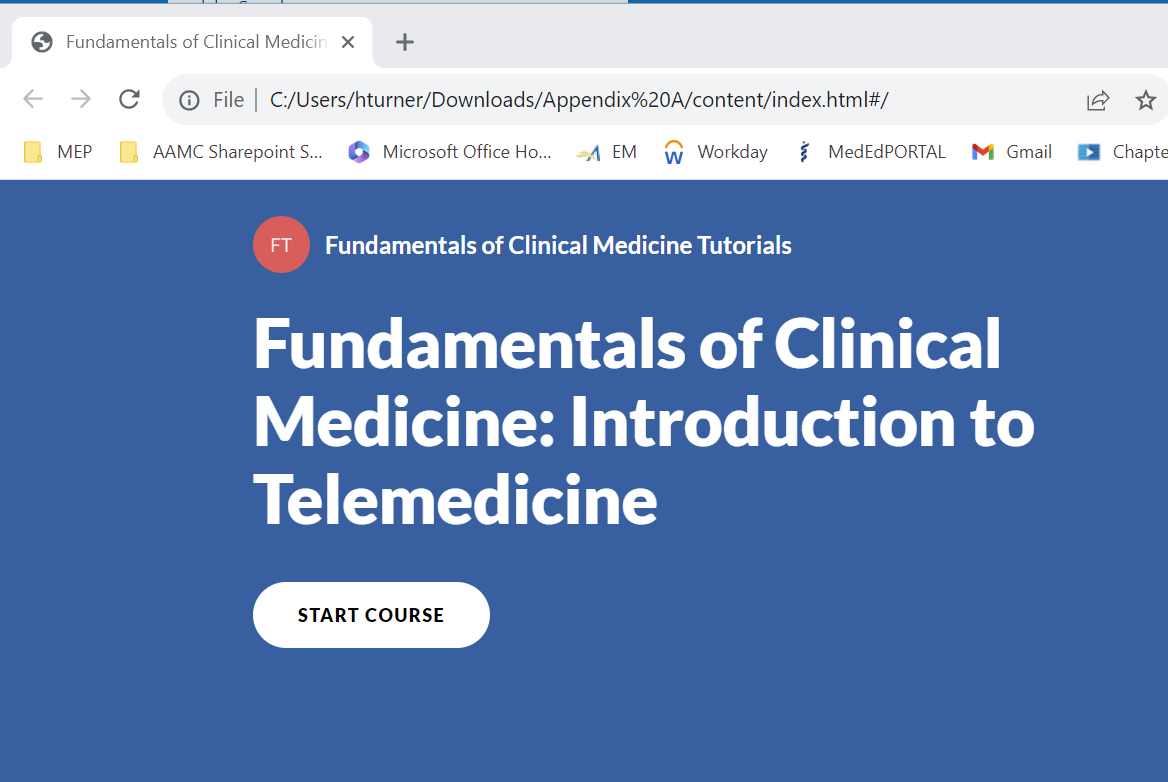


Please email [mededportal@aamc.org](mailto:mededportal@aamc.org) with any questions about accessing submission files.
