# Supplementary material for: An Interactive, Asynchronous Intimate Partner Violence Module for Medical Students: Improving Preparedness, Confidence, and Knowledge
Source: MedEdPORTAL. 2026 Jul 14;22:11618. doi: 10.15766/mep_2374-8265.11618 (PMC13364887; doi:10.15766/mep_2374-8265.11618)
Supplement: Supplementary file 1 — IPV Articulate Module FolderIPV Pre- and Postmodule Survey.docx [file mep_2374-8265.11618-s001.zip › A. IPV Articulate Module Folder/START HERE.html]

Intimate Partner Violence
