# Supplementary material for: An Interactive, Asynchronous Intimate Partner Violence Module for Medical Students: Improving Preparedness, Confidence, and Knowledge
Source: MedEdPORTAL. 2026 Jul 14;22:11618. doi: 10.15766/mep_2374-8265.11618 (PMC13364887; doi:10.15766/mep_2374-8265.11618)
Supplement: Supplementary file 1 — IPV Articulate Module FolderIPV Pre- and Postmodule Survey.docx [file mep_2374-8265.11618-s001.zip › B. IPV Pre- and Postmodule Survey.docx]

IPV Pre- and Post- Module Survey:

Hello,

The Department of Psychiatry has created a module on the topic of Intimate Partner Violence (IPV). This course will take approximately 60 minutes to complete. The objectives of this course are to learn what IPV is, the epidemiology and risk factors of IPV, how to assess, ask about, and document IPV, mandated reporting surrounding IPV, and treatment and resources for victims of IPV. This module will not impact your academic standing.

There will be a mandatory pre and post knowledge-based survey for this course. An additional optional survey containing demographic information and questions regarding your comfort/attitude towards IPV will also be available. As part of this study, researchers will be collecting data that is protected by the Federal Educational Records Protection Act (FERPA). The records will be collected as part of the research study of the eﬀectiveness of the Psychotherapy Clerkship Module. These records will include the module required knowledge portion of the pre/post test of the module as well as the optional demographic and comfort/attitudes portion of the module survey. These records will be collected once during the clerkship, and disclosed to only to the researchers of the study. The data will be de-identified prior to analysis. Please sign your name below if you grant permission to utilize your de-identified data in this study. If some of the material or questions make you feel uncomfortable you may stop the course or surveys at any time. Other coursework re: IPV will be available. Medical student participation in this study has been approved by the Associate Dean of Medical Education. If you have questions, concerns please reach out to Dr. Beth Harper (Bethany.harper@wright.edu) or Dr. Larrilyn Grant (larrilyn.grant@wright.edu).

Thank you,

Dr. Harper and Dr. Grant

Please sign below to consent for your de-identified data to be used for research purposes.

×

SIGN HERE

1. What is your age
   - Younger than 20 years old
   - 20-24 years old
   - 25-29 years old
   - 30-34 years old
   - 35-39 years old
   - 40 years old or older
   - Prefer not to respond
2. What is your gender identity
   - Male
   - Female
   - Non-binary
   - Transgender male
   - Transgender female
   - Agender//I don’t identify with a gender
   - Gender not listed. My gender is: ______
   - Prefer not to state
3. What is your intended specialty?
   - Internal medicine
   - Family medicine
   - Pediatrics
   - Psychiatry
   - Emergency medicine
   - Surgery
   - Obstetrics/Gynecology
   - Other (specify) ____
   - Prefer not to respond
4. Please select which best describes how prepared you feel to perform the following:
   - Questions to ask to identify IPV
     - Not prepared
     - Minimally
     - Slightly
     - Moderately
     - Fairly well
     - Well
     - Quite well
   - Signs of IPV
     - Not prepared
     - Minimally
     - Slightly
     - Moderately
     - Fairly well
     - Well
     - Quite well
   - Appropriately responding to disclosures of abuse
     - Not prepared
     - Minimally
     - Slightly
     - Moderately
     - Fairly well
     - Well
     - Quite well
   - Relationship between IPV and pregnancy
     - Not prepared
     - Minimally
     - Slightly
     - Moderately
     - Fairly well
     - Well
     - Quite well
   - Assess and IPV victim’s readiness to change
     - Not prepared
     - Minimally
     - Slightly
     - Moderately
     - Fairly well
     - Well
     - Quite well
   - Providing referral sources to victims of IPV
     - Not prepared
     - Minimally
     - Slightly
     - Moderately
     - Fairly well
     - Well
     - Quite well
   - Help IPV victim create a safety plan
     - Not prepared
     - Minimally
     - Slightly
     - Moderately
     - Fairly well
     - Well
     - Quite well
   - Document IPV history and physical examination findings in a patient’s chart
     - Not prepared
     - Minimally
     - Slightly
     - Moderately
     - Fairly well
     - Well
     - Quite well
   - Fulfill state reporting requirements for IPV
     - Not prepared
     - Minimally
     - Slightly
     - Moderately
     - Fairly well
     - Well
     - Quite well
   - Fulfill state reporting requirements for Child Abuse
     - Not prepared
     - Minimally
     - Slightly
     - Moderately
     - Fairly well
     - Well
     - Quite well
   - Fulfill state reporting requirements for Elder Abuse
     - Not prepared
     - Minimally
     - Slightly
     - Moderately
     - Fairly well
     - Well
     - Quite well
5. What is the strongest single risk factor for becoming a victim of intimate partner violence?
   - Age (<30 years old)
   - Partner abuses alcohol/drugs
   - Gender-female
   - Family history of abuse
   - I don’t know
6. Which of the following is generally true about batterers?
   - They have trouble controlling their anger
   - They use violence as a means of controlling their partners
   - They are violent because they drink or use drugs
   - They pick fights with anyone
7. Which of the following are the warning signs that a patient may have been abused by their partner? (Check all that apply)
   - Chronic unexplained pain
   - Anxiety
   - Substance Abuse
   - Frequent injuries
   - Depression
8. Which of the following are reasons an IPV victim may not be able to leave a violent relationship? (Select all that apply)
   - Fear of retribution
   - Financial dependence of the perpetrator
   - Religious beliefs
   - Children’s needs
   - Love for one’s partner
   - Isolation
9. Which of the following are the most appropriate ways to ask a patient about IPV?
   - "Are you a victim of intimate partner violence?"
   - "Has your partner ever hurt or threatened you"
   - "Have you ever been afraid of your partner?"
   - "Has your partner ever hit or hurt you?"
10. Which of the following is/are generally true? (Select all that apply)
    - There are common, non-injury presentations of abused patients
    - There are behavioral patterns in couples that may indicate IPV
    - Specific areas of the body are most often targeted in IPV cases
    - There are common injury patterns associated with IPV
    - Injuries in different stages of recovery may indicate abuse
11. Please match the following descriptions of the behaviors and feelings of patients with a history of IPV with the appropriate stage of change
    - Begins making plans for leaving the abusive partner
      - Precontemplation
      - Contemplation
      - Preparation
      - Action
      - Maintenance
    - Denies there is a problem
      - Precontemplation
      - Contemplation
      - Preparation
      - Action
      - Maintenance
    - Continues changing behaviors
      - Precontemplation
      - Contemplation
      - Preparation
      - Action
      - Maintenance
    - Begins thinking the abuse is not their own fault
      - Precontemplation
      - Contemplation
      - Preparation
      - Action
      - Maintenance
    - Obtains order(s) for protection
      - Precontemplation
      - Contemplation
      - Preparation
      - Action
      - Maintenance
12. For each of the following statements, please indicate your response on the scale from "Strongly Disagree" (1) to "Strongly Agree" (5).
    - If an IPV victim does not acknowledge the abuse, there is very little that I can do to help.
      - Strongly disagree
      - Somewhat disagree
      - Neither agree or disagree
      - Somewhat agree
      - Strongly agree
    - I will ask all new patients about abuse in their relationships.
      - Strongly disagree
      - Somewhat disagree
      - Neither agree or disagree
      - Somewhat agree
      - Strongly agree
    - I do not have sufficient training to assist individuals in addressing situations of IPV.
      - Strongly disagree
      - Somewhat disagree
      - Neither agree or disagree
      - Somewhat agree
      - Strongly agree
    - Patients who abuse alcohol or other drugs are likely to have a history of IPV.
      - Strongly disagree
      - Somewhat disagree
      - Neither agree or disagree
      - Somewhat agree
      - Strongly agree
    - Victims of abuse have the right to make their own decisions about whether hospital staff should intervene.
      - Strongly disagree
      - Somewhat disagree
      - Neither agree or disagree
      - Somewhat agree
      - Strongly agree
    - I feel comfortable discussing IPV with my patients.
      - Strongly disagree
      - Somewhat disagree
      - Neither agree or disagree
      - Somewhat agree
      - Strongly agree
    - I don't have the necessary skills to discuss abuse with an IPV victim who is female.
      - Strongly disagree
      - Somewhat disagree
      - Neither agree or disagree
      - Somewhat agree
      - Strongly agree
    - I don't have the necessary skills to discuss abuse with an IPV victim who is male.
      - Strongly disagree
      - Somewhat disagree
      - Neither agree or disagree
      - Somewhat agree
      - Strongly agree
    - I don't have the necessary skills to discuss abuse with an IPV victim who is gender diverse.
      - Strongly disagree
      - Somewhat disagree
      - Neither agree or disagree
      - Somewhat agree
      - Strongly agree
    - Health care providers do not have the time to assist patients in addressing IPV.
      - Strongly disagree
      - Somewhat disagree
      - Neither agree or disagree
      - Somewhat agree
      - Strongly agree
    - If a patient refuses to discuss the abuse, staff can only treat the patient's injuries
      - Strongly disagree
      - Somewhat disagree
      - Neither agree or disagree
      - Somewhat agree
      - Strongly agree
    - Health care providers have a responsibility to ask all patients about IPV.
      - Strongly disagree
      - Somewhat disagree
      - Neither agree or disagree
      - Somewhat agree
      - Strongly agree
    - Victims of abuse often have valid reasons for remaining in the abusive relationship.
      - Strongly disagree
      - Somewhat disagree
      - Neither agree or disagree
      - Somewhat agree
      - Strongly agree
    - Screening for IPV is likely to offend those who are screened.
      - Strongly disagree
      - Somewhat disagree
      - Neither agree or disagree
      - Somewhat agree
      - Strongly agree
    - Health care providers do not have the knowledge to assist patients in addressing IPV.
      - Strongly disagree
      - Somewhat disagree
      - Neither agree or disagree
      - Somewhat agree
      - Strongly agree
