# Supplementary material for: An Interactive, Asynchronous Intimate Partner Violence Module for Medical Students: Improving Preparedness, Confidence, and Knowledge
Source: MedEdPORTAL. 2026 Jul 14;22:11618. doi: 10.15766/mep_2374-8265.11618 (PMC13364887; doi:10.15766/mep_2374-8265.11618)
Supplement: Supplementary file 1 — IPV Articulate Module FolderIPV Pre- and Postmodule Survey.docx [file mep_2374-8265.11618-s001.zip › _Educational Summary Report_11618.pdf]

MedEdPORTAL
THE AAMC JOURNAL OF TEACHING AND LEARNING RESOURCES

Original Publication

Open Access

An Interactive, Asynchronous Intimate Partner Violence Module for Medical Students: Improving Preparedness, Confidence, and Knowledge

Victoria Ivy Petsinger*,†, Sugapradha Saravanan†, Rahul Manne, Ramzi W. Nahhas, PhD, Bethany Harper, MD, Larrilyn Grant, MD

*Corresponding author: petsinger.2@wright.edu
†Co-primary author

Abstract

Introduction: Intimate partner violence (IPV) is a cyclical pattern of behavior that can cause physical, psychological, and/or sexual harm with significant risks, including posttraumatic stress disorder. The primary aim of IPV is to establish or maintain power over the partner. While many organizations recommend screening for IPV, there is limited exposure to IPV in medical school curricula. This critical gap represents opportunity for further education. Thus, an interactive module on IPV was created for medical students on their psychiatry clerkship to teach skills in assessment, treatment planning, and resources. This study evaluates the module's effectiveness and medical students' readiness in addressing IPV in clinical settings. **Methods:** The module provides a structured overview of IPV, including epidemiology, barriers to seeking help, and screening approaches. It incorporates video-based clinical scenarios, allowing learners to observe and practice how to recognize and respond to IPV in real-world settings. Pre- and postmodule surveys were administered to assess changes in knowledge, confidence, and preparedness in addressing IPV. Individual item scores were summed within each domain, and pre- and postmodule total scores were compared using paired *t* tests. **Results:** Data were collected from 122 third-year medical students. Students showed statistically significant improvements in knowledge and reported higher confidence and preparedness ($P < .001$) after completing the module. More students correctly identified risk factors, felt adequately trained, and were more comfortable discussing IPV. **Discussion:** The IPV module improved students' knowledge, confidence, and preparedness in addressing IPV. Future studies should assess long-term retention and its impact on clinical practice.

Keywords
Intimate Partner Violence, Trauma-Informed Care, Asynchronous Learning, Psychiatry

Educational Objectives

By the end of the activity, learners will be able to:

1. Demonstrate increased knowledge of the health complications and epidemiologic factors associated with intimate partner violence.
2. Report increased comfort with screening for and discussing intimate partner violence in clinical settings.
3. Report increased preparedness to recognize and respond to patients experiencing intimate partner violence.

Citation:
Petsinger VI, Saravanan S, Manne R, Nahhas RW, Harper B, Grant L. An interactive, asynchronous intimate partner violence module for medical students: improving preparedness, confidence, and knowledge. *MedEdPORTAL*. 2026;22:11618.
https://doi.org/10.15766/mep_2374-8265.11618

Introduction

Intimate partner violence (IPV) remains a pervasive public health crisis in the United States (US), affecting individuals across all demographics.[1] According to the US Centers for Disease Control and Prevention[1] (CDC), approximately 1 in 4 women and 1 in 10 men report experiencing IPV in their lifetime, which may include physical, sexual, psychological, or economic abuse. IPV impacts one's emotional, psychological, physical, and financial well-being, while also placing a substantial burden on health care systems and educational institutions responsible for training future professionals. According to Peterson et al.,[2] the estimated IPV lifetime cost among approximately 43 million US adults with a history of experiencing IPV was nearly $3.6 trillion.

Survivors of IPV frequently endure depression, anxiety, posttraumatic stress disorder (PTSD), and suicidal ideation.[3] A systematic review and meta-analysis identified strong associations between psychological violence and mental health disorders, with particularly large effect sizes for depression and

Copyright © 2026 Petsinger et al. This is an open-access publication distributed under the terms of the Creative Commons Attribution license.

PTSD among women.[4] These effects often persist long after the abusive relationship ends, affecting survivors' ability to function in daily life and maintain relationships.[4]

IPV survivors may also suffer acute injuries such as bruises, fractures, and head trauma, as well as chronic conditions including gastrointestinal disorders, migraines, and chronic pain.[5] IPV during pregnancy is associated with heightened risk of miscarriage, preterm labor, and fetal injury, often resulting in long-term medical complications and increased health care utilization.[6]

Economic abuse, a frequently overlooked aspect of IPV, can be equally debilitating. Abusers may restrict survivors' access to financial resources, sabotage employment opportunities, or accumulate debt in the victim's name, leading to prolonged economic dependence. Additionally, IPV contributes to substantial societal costs through lost productivity and increased health care expenditures, with the CDC estimating the economic burden in the billions each year.[7]

Despite the prevalence and impact, IPV remains insufficiently addressed in medical, nursing, and graduate health education. Studies show that even after formal training, health care professionals often feel ill-prepared to identify and respond to IPV.[8] Medical students similarly report limited training and low confidence in managing IPV cases. A 2019 study investigated 204 health care providers, 27 of which were social and behavioral health workers. These health care professionals specifically required more preparation, knowledge, and survivor understanding regarding IPV training. Even with the extra training, these 27 reported the lowest screening rates of IPV compared to other health care personnel groups.[9] This lack of confidence was also apparent in a study that adapted the Physician Readiness to Manage Intimate Partner Violence Survey (PREMIS) for pharmacy students. A total of 144 surveys were collected, and the results showed low levels of knowledge in recognizing and treating IPV.[10] These educational deficiencies hinder health care professionals' ability to identify and support IPV survivors effectively.

Most existing IPV curricula for medical students are short term (less than 1 academic year) and rely primarily on formal lectures and standardized patients as delivery methods.[8] Standardized patient interventions have shown to be effective, with 1 study demonstrating significant improvements in comfort recognizing IPV signs and knowledge of resources.[9] However, these approaches require substantial resources and faculty time, which are commonly cited as the biggest implementation barriers.[9] Medical schools in Australia report a median time of only 3-6 contact hours for IPV education, with time constraints and resource shortages as key barriers.[10] Existing curricula also demonstrate significant gaps in addressing treatment planning and resource navigation. A scoping review found that most curricula taught risk factors and identification of IPV but lacked comprehensive coverage of management and intervention strategies.[8] This is problematic because screening alone without intervention does not improve outcomes. Rather, effective approaches require ongoing support services, counseling, and connection to community resources. Additionally, published curricula show great variation in assessment methods without consistent validated tools for measuring effectiveness, making it difficult to establish competency standards.[8]

This module is novel in several key areas. It represents an asynchronous, interactive module format that has also been updated to meet federal accessibility standards, ensuring usability for a broad range of learners. While most published curricula use formal lectures (resource-intensive) or standardized patients (expensive and time-limited), asynchronous e-learning modules offer scalability and flexibility that address the primary implementation barrier of limited time in medical education.

The use of an interactive asynchronous module aligns with several educational principles. Interactive modules allow students to engage actively with content rather than passively receiving information. Evidence shows that interventions based on role-plays and feedback are more effective than lectures alone for communication skills development. Video-recorded peer role-play with standardized checklists has demonstrated effectiveness in developing communication competencies among first-year medical students, with 87% agreeing that it was an effective learning method.[10] Asynchronous formats support learner autonomy and self-paced engagement, which is particularly important for sensitive topics where students may need time for reflection and emotional processing. Medical students report that trauma-informed teaching strategies, including preparedness for teaching and appropriate debriefing, promote psychological safety when learning about sensitive topics like domestic violence.[11] While some evidence suggests synchronous learning may reduce cognitive load compared to asynchronous formats, asynchronous modules allow students to pause, reflect, and revisit complex content, which is particularly valuable for learning communication skills around sensitive topics that may trigger emotional responses.

The purpose of this module is to provide medical students with a structured framework for understanding IPV. The module

Copyright © 2026 Petsinger et al. This is an open-access publication distributed under the terms of the Creative Commons Attribution license.

highlights key risk factors that contribute to IPV, outlines its harmful effects on survivors, and describes clinical signs and symptoms that may indicate abuse. It also guides learners through evidence-based steps for supporting survivors and connecting them to appropriate resources and care. This module is oriented to the baseline material of dealing with common presentations of IPV and does not address the more complex, nuanced presentations.

Currently, there are several free training resources available that are appropriate for third-year medical students learning about IPV. The World Health Organization (WHO) offers a curriculum titled “Caring for Women Subjected to Violence: A WHO Curriculum for Training Health Care Providers.”[11] This comprehensive curriculum is specifically designed for health care provider training and includes structured modules that can be adapted for self-directed learning. The Futures Without Violence Association offers free educational videos on the CUES approach for IPV (Confidentiality, Universal Education and Empowerment, Support).[12] The Agency for Healthcare Research and Quality also offers a comprehensive table of IPV screening instruments with items, scoring, and citations, which can serve as self-study resources for learning validated assessment tools.[13] Despite the availability of these resources, there remains a gap for interactive, case-based IPV training in medical education. Our asynchronous module addresses this through clinical decision-making and patient-centered communication.

Methods

We developed an asynchronous, self-paced, online module as part of the third-year psychiatry clerkship at a midwestern medical school. Our team, comprising 2 psychiatrists specializing in medical education and a group of psychiatry residents and medical students interested in medical education, used Articulate software to create the course. Literature supports the use of realistic case-based scenarios to promote critical thinking, problem analysis, and reflective decision-making in online learning environments. To that end, we adapted a clinical case originally used in a live session on IPV and modified it for self-guided digital learning.[11,14,15] The module takes approximately 1 hour to complete.

The curriculum is centered around a longitudinal case of a 21-year-old patient experiencing IPV across outpatient, emergency, inpatient psychiatry, and follow-up settings. Through this case, learners engage with core concepts such as IPV definitions, trauma-informed interviewing, clinical and legal considerations, barriers to care, case conceptualization, and safety planning. The module incorporates interactive components to reinforce learning, including branched patient interview scenarios, multiple choice questions, and sorting/matching exercises for topics such as identifying risk factors, warning signs, and stages of abuse. Videos throughout the module portray patient–provider interactions that highlight nonverbal cues and clinical decision-making. Additional activities include differential diagnosis exercises, reflective prompts (eg, countertransference), and exposure to community resources, including an audio recording of a hotline phone call with an advocate. Embedded expert commentary is provided throughout to emphasize key teaching points. Appendix A contains the full course. This course has been updated to meet federal accessibility standards.

All third-year medical students in the 2024-2025 academic year completed the course during the fourth week of their psychiatry clerkship. Students had no prior required IPV-specific training, but they had completed standard clerkship orientation and initial inpatient exposure by that time. No special audiovisual equipment or in-person facilitation was needed for implementation.

We assessed learner outcomes using pre- and postmodule surveys (Appendix B) adapted from the validated PREMIS.[16] Terminology throughout the manuscript was revised, when possible, to reflect trauma-informed language. Terms such as “victim” and “batterer” were kept only when referencing the validated PREMIS instrument and corresponding survey items. We obtained informed consent to use de-identified survey data for research purposes and provided students with information about Family Educational Records Protection Act (FERPA) protections and the voluntary nature of participation (see Appendix B).

We administered the survey electronically before and after module completion. Each Likert-scale item was converted to a numerical value, beginning at 0. We reverse-coded negatively worded items so that higher scores reflected greater agreement in the intended direction. One knowledge item was excluded due to poor contribution to internal consistency. Item-level analysis demonstrated that removing a question related to appropriate IPV screening practices increased Cronbach’s alpha from 0.49 at each time (pre and post) to 0.568 (pre) and 0.583 (post), and it was therefore excluded from the total knowledge score. We calculated total scores for preparation (8 items, 0-6 scale), knowledge (26 items, scored 1 if correct), and agreement (15 items, 0-4 scale). Total scores were then rescaled so that each was in the range 0 to 100. Cronbach’s alpha indicated strong internal consistency for preparation ($\alpha = 0.919$ pre, 0.951 post),

Copyright © 2026 Petsinger et al. This is an open-access publication distributed under the terms of the Creative Commons Attribution license.

moderate for agreement ($\alpha = 0.692$ pre, 0.761 post), and lower for knowledge ($\alpha = 0.568$ pre, 0.583 post).

We computed descriptive statistics (means, standard deviations, frequencies, proportions) for each item and total scores at both time points.[17] Individual ordinal items were compared between times using a cumulative link mixed model (CLMM); individual binary items were compared using McNemar's test; and each total was compared between times using a paired-samples *t* test.

We received IRB approval for this study from Wright State University (IRB-2023-411), and the study was determined to be exempt.

We conducted data processing and analysis in R version 4.3.2.[18] We used the ordinal package to fit CLMMs.[19] All tests were 2-tailed, with α set at 0.05.

Results

A total of 122 out of 131 third-year medical students (93.1%) consented to participate in the study and completed both the pre- and postmodule surveys. Most participants were between 25 and 29 years old, with a majority identifying as female.

Knowledge Outcomes

Student knowledge showed a small but statistically significant increase following the module (mean difference = 2.77, 95% CI = 1.45, 4.10, $P < .001$). Correct identification of "gender female" as the strongest IPV risk factor increased from 27.9% to 54.9% ($P < .001$), and recognition that batterers use violence to control their partners increased from 83.6% to 94.3% ($P < .001$). Other items demonstrated little change, including recognition of abuse signs (from 82.0% to 82.8%, $P = 1.000$) and reasons survivors may not leave (from 91.0% to 91.8%, $P = 1.000$). For the multiselect item assessing general knowledge of IPV, the proportion of students selecting all correct options increased from 66.4% to 73.8% ($P = .110$). Full results are summarized in Table 1.

Perceptions and Confidence

Perceptions and confidence improved significantly following the module (mean difference in total score = 12.04, 95% CI = 10.32, 13.75, $P < .001$), reflecting greater comfort and more positive attitudes toward identifying and responding to IPV.

Students demonstrated important shifts in perceptions: strong disagreement with "If an IPV victim does not acknowledge the abuse, there is very little that I can do to help" increased from 20.5% to 35.2% ($P = .002$). Confidence in screening also improved—those strongly agreeing with "I will ask all new patients about abuse in their relationships" nearly doubled, increasing from 24.6% to 48.4% ($P < .001$). Perceived training gaps decreased, with the proportion of students disagreeing that they lacked sufficient training rising from 27.0% to 74.6% ($P < .001$).

Comfort discussing IPV also grew markedly. Agreement with the statement "I feel comfortable discussing IPV with my patients" increased from 32.0% to 84.4% ($P < 0.001$). Disagreement that students lacked skills to discuss abuse with a female, male, or gender-diverse individual rose substantially (female 43.4% to 82.0%, male 37.7% to 77.0%, and gender diverse 37.7% to 74.6%; $P < .001$ each).

Recognition of provider responsibility also increased. Strong agreement that "Health care providers have a responsibility to ask all patients about IPV" rose from 39.3% to 59.8% ($P < .001$), whereas agreement that providers lack the time or knowledge to help decreased significantly (4.9% to 0%, $P = .018$; 6.6% to 2.4%, $P = .005$; respectively).

Empathy and validation improved as well, with greater agreement that survivors may have valid reasons for remaining in abusive

Table 1. Pre- and Postmodule Knowledge Item Performance ($N = 122$)

Knowledge Item[a]	Premodule (%)[b]	Postmodule (%)[b]	*P*[c]
Identified "gender female" as strongest risk factor for IPV	27.9	54.9	<.001
Identified "batterers use violence to control partners"	83.6	94.3	.004
Correctly selected all warning signs of IPV[d]	82.0	82.8	1.000
Identified all valid reasons victims may not leave relationships[d]	91.0	91.8	1.000
Correctly matched all stages of change with patient behavior[d]	73.8	77.9	.441
Selected only the correct ways to ask about IPV[d]	2.5	0	.248
Selected all true statements about IPV patterns[d]	66.4	73.8	.110

Abbreviation: IPV, intimate partner violence.
[a]Terminology throughout the article was revised, when possible, to reflect trauma-informed language. Terms such as "victim" and "batterer" were kept only when referencing the validated Physician Readiness to Manage Intimate Partner Violence Survey (PREMIS) instrument and corresponding survey items.
[b]The values are the percentage of students answering each item correctly.
[c]*P* values are from McNemar's test.
[d]For multiselect/matching items, "correct" required selecting all correct options and no incorrect options.

Copyright © 2026 Petsinger et al. This is an open-access publication distributed under the terms of the Creative Commons Attribution license.

Table 2. Pre- and Postmodule Perceptions and Confidence Item Performance ($N = 122$)

	Response, No. (%)					
Item[a]	Strongly Disagree	Somewhat Disagree	Neither Agree nor Disagree	Somewhat Agree	Strongly Agree	*P*
**If an IPV victim does not acknowledge the abuse, there is very little that I can do to help.**						.002
Pre	25 (20.5)	64 (52.5)	16 (13.1)	17 (13.9)	0 (0)	
Post	43 (35.2)	56 (45.9)	7 (5.7)	15 (12.3)	1 (0.8)	
**I will ask all new patients about abuse in their relationships.**						<.001
Pre	2 (1.6)	9 (7.4)	20 (16.4)	61 (50.0)	30 (24.6)	
Post	2 (1.6)	5 (4.1)	10 (8.2)	46 (37.7)	59 (48.4)	
**I do not have sufficient training to assist individuals in addressing situations of IPV.**						<.001
Pre	7 (5.7)	26 (21.3)	31 (25.4)	43 (35.2)	15 (12.3)	
Post	28 (23.0)	63 (51.6)	17 (13.9)	14 (11.5)	0 (0)	
**I feel comfortable discussing IPV with my patients.**						<.001
Pre	6 (4.9)	33 (27.0)	44 (36.1)	31 (25.4)	8 (6.6)	
Post	0 (0.0)	3 (2.5)	16 (13.1)	76 (62.3)	27 (22.1)	
**Victims of abuse often have valid reasons for remaining in the abusive relationship.**						.050
Pre	3 (2.5)	4 (3.3)	32 (26.2)	59 (48.4)	24 (19.7)	
Post	5 (4.1)	5 (4.1)	17 (13.9)	65 (53.3)	30 (24.6)	

Abbreviation: IPV, intimate partner violence.
[a]Terminology throughout the article was revised, when possible, to reflect trauma-informed language. Terms such as "victim" and "batterer" were kept only when referencing the validated Physician Readiness to Manage Intimate Partner Violence Survey (PREMIS) instrument and corresponding survey items.

relationships (68.1% to 77.9%, $P = .050$). Students also became less concerned that screening would offend patients as strong disagreement with this concern increased (11.5% to 22.1%), although this change was not statistically significant ($P = .149$).

Examples of 5 items in this category with agreement levels pre- and postmodule and *P* values are provided in Table 2.

Preparedness
Students reported a large and statistically significant increase in preparedness to address IPV following the module (mean difference = 29.27, 95% CI = 26.17, 32.36, $P < .001$). As shown in the Figure, almost every student increased their total score postmodule. Improvements were observed across all domains, including identifying risk factors and signs of IPV, responding to disclosures, understanding IPV in pregnancy, providing referrals, developing safety plans, and documenting IPV in the medical record. Collectively, these findings indicate broad gains in students' confidence and skills in managing IPV in clinical

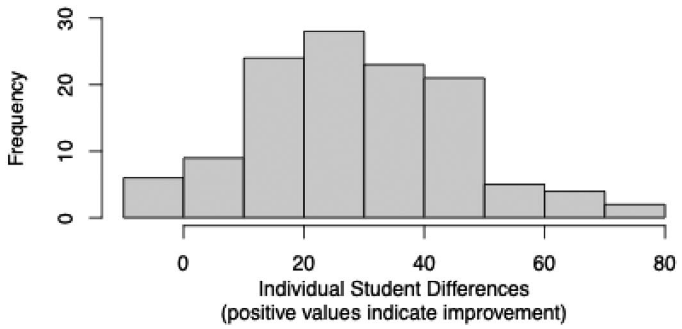

Figure. Individual student change in total preparedness score (postmodule score − premodule score, each rescaled to 0-100; positive values indicate improvement) (mean = 29.3, *SD* = 17.3, 95% CI = 26.2, 32.4).

settings. Examples of 5 items in this category with preparedness levels pre- and postmodule and *P* values are summarized in Table 3.

Discussion

This study demonstrates that our IPV learning module produced statistically significant improvements in knowledge, perceptions and confidence, and preparedness among third-year medical students. The largest gains were observed in preparedness, with more modest improvements in perceptions and confidence, and the smallest change in knowledge. These findings suggest that while students may enter clinical training with baseline knowledge about IPV, structured educational approaches can meaningfully enhance their readiness to apply IPV-related skills in clinical practice.

These results align with prior literature demonstrating that IPV education improves learner knowledge, attitudes, and readiness, while also highlighting ongoing challenges in translating knowledge into clinical practice.[8] Given the limited coverage of IPV training in medical education, this asynchronous module addresses a critical educational need by combining conceptual knowledge and practical clinical strategies in a scalable, flexible, and accessible format for resource-limited curricula.

Knowledge
The module improved students' factual understanding of IPV, including recognition of epidemiologic risk factors (eg, female gender), patterns of abusive behavior and injury patterns. However, some items showed little change due to high baseline scores, whereas others revealed gaps in instruction. For example, performance on a multiselect item assessing appropriate ways to

Copyright © 2026 Petsinger et al. This is an open-access publication distributed under the terms of the Creative Commons Attribution license.

Table 3. Pre- and Postmodule Preparedness Item Performance (*N* = 122)

Item[a]	Response, No. (%)							*P*
	Not Prepared	Minimally	Slightly	Moderately	Fairly Well	Well	Quite Well	
**Questions to ask to identify IPV**								<.001
Pre	5 (4.1)	24 (19.7)	35 (28.7)	37 (30.3)	18 (14.8)	3 (2.5)	0 (0)	
Post	0 (0)	1 (0.8)	5 (4.1)	40 (32.8)	40 (32.8)	28 (23.0)	8 (6.6)	
**Signs of IPV**								<.001
Pre	3 (2.5)	12 (9.8)	34 (27.9)	46 (37.7)	21 (17.2)	5 (4.1)	1 (0.8)	
Post	0 (0)	0 (0)	2 (1.6)	26 (21.3)	47 (38.5)	35 (28.7)	12 (9.8)	
**Providing referral sources to victims of IPV**								<.001
Pre	19 (15.6)	28 (23.0)	35 (28.7)	27 (22.1)	10 (8.2)	3 (2.5)	0 (0)	
Post	1 (0.8)	4 (3.3)	13 (10.7)	31 (25.4)	35 (28.7)	28 (23.0)	10 (8.2)	
**Help IPV victim create a safety plan**								<.001
Pre	21 (17.2)	27 (22.1)	34 (27.9)	28 (23.0)	9 (7.4)	3 (2.5)	0 (0)	
Post	0 (0)	4 (3.3)	11 (9.0)	37 (30.3)	30 (24.6)	29 (23.8)	11 (9.0)	
**Document IPV history and physical examination findings in a patient's chart**								<.001
Pre	19 (15.6)	27 (22.1)	33 (27.0)	26 (21.3)	11 (9.0)	6 (4.9)	0 (0)	
Post	0 (0)	3 (2.5)	10 (8.2)	31 (25.4)	36 (29.5)	31 (25.4)	11 (9.0)	

Abbreviation: IPV, intimate partner violence.
[a]Terminology throughout the article was revised, when possible, to reflect trauma-informed language. Terms such as "victim" and "batterer" were kept only when referencing the validated Physician Readiness to Manage Intimate Partner Violence Survey (PREMIS) instrument and corresponding survey items.

ask about IPV was very poor both before and after the module, suggesting the need for clearer teaching or question revision. These mixed results highlight both the strengths of the module in reinforcing foundational knowledge and the need for more explicit instruction around nuanced clinical communication skills, such as trauma-informed questioning, through interactive or clinically integrated approaches.

Perceptions and Confidence

Students reported increased confidence in screening for and discussing IPV, with marked improvements in their willingness to ask about abuse and their comfort interacting with survivors across gender identities. These findings suggest that the module helped normalize IPV as a clinical topic and reinforced provider responsibility. At the same time, perceptions of systemic barriers (eg, limited provider time) shifted only modestly, underscoring structural challenges that extend beyond individual training. While self-reported confidence increased, this module represents an introductory educational experience, and we cannot conclude that these gains translate to clinical competence. Without supervised clinical encounters, students may overestimate their readiness to manage complexities of IPV care, including safety considerations, longitudinal care, and family dynamics.

Preparedness

Preparedness showed the greatest improvement, particularly in safety planning, referrals, and documenting IPV in the medical record. These findings suggest that interactive, case-based learning may be effective in supporting the development of actionable IPV-related clinical skills. However, preparedness was assessed through self-report rather than observation. Future curricula should incorporate supervised clinical practices, such as standardized patient encounters, to evaluate competency directly.

Implications

These findings have important implications for medical education. While brief educational modules can improve student knowledge, perceptions, confidence, and preparedness, they should be framed as foundational experiences within a broader, longitudinal curriculum. Integrating IPV training across multiple clerkships and incorporating supervised clinical encounters can support retention and promote clinical application. Additionally, addressing perceived systemic barriers, such as time constraints, may require institutional and curricular support within clinical environments.

Limitations

This study has several limitations. First, knowledge retention was not assessed longitudinally; long-term impact remains unknown. Second, outcomes were based on self-report, which may not reflect true clinical performance. Finally, results from a single cohort at 1 institution limit generalizability.

Future Directions

Future research should include multiple institutions and cohorts, with longitudinal assessments to evaluate knowledge retention and skill application over time. Expanding IPV training into preclerkship curricula and reinforcing this during clerkships could ensure progressive skill development. Preclinical training can emphasize IPV identification, epidemiology, and case-based discussions. Extending this training beyond psychiatry to other

Copyright © 2026 Petsinger et al. This is an open-access publication distributed under the terms of the Creative Commons Attribution license.

clerkships would promote interprofessional engagement and strengthen the health care system's ability to respond to IPV comprehensively. The module can be tailored for specialty-focused scenarios that would involve IPV. Incorporating standardized patient encounters and observed history-taking exercises would provide opportunities to assess competency directly.

Future iterations of the module could incorporate additional scenarios involving different cultural and language considerations (eg, addressing IPV in non–English-speaking patients and incorporating different cultural barriers to IPV so that a larger number of patients can be offered resources), overt aggression, substance involvement, and child protection considerations to further reflect the complexity of IPV in clinical practice. Future research can focus on the impact of incorporating such changes and assessing these skills at various points in time to determine the long-term impact of the IPV training and retention of skills.

Appendices

A. IPV Articulate Module Folder

B. IPV Pre- and Postmodule Survey.docx

All appendices are peer reviewed as integral parts of the Original Publication.

Victoria Ivy Petsinger: Medical Student, Wright State University Boonshoft School of Medicine; ORCID: https://orcid.org/0009-0008-7849-5716

Sugapradha Saravanan: Medical Student, Wright State University Boonshoft School of Medicine

Rahul Manne: Medical Student, Wright State University Boonshoft School of Medicine

Ramzi W. Nahhas, PhD: Associate Professor, Wright State University Boonshoft School of Medicine Department of Population and Public Health Sciences, Department of Psychiatry

Bethany Harper, MD: Associate Professor, Wright State University Boonshoft School of Medicine Department of Psychiatry

Larrilyn Grant, MD: Assistant Professor, Wright State University Boonshoft School of Medicine Department of Psychiatry

Acknowledgments
Thank you to Dr. Bria Williams, Dr. Basir Hussaini, Dr. Simone Stinnette, Dr. Allison Chen, Dr. Azl Saeed, Dr. Erika Nguyen, and Dr. Genevieve Miller for prior work on this project.

Disclosures
None to report.

Funding/Support
None to report.

Prior Presentation
Petsinger I, Saravanan S, Manne R, Nahhas RW, Harper B. Evaluating the impact of an interactive intimate partner violence module on medical student preparedness, knowledge, and confidence. Poster presented at: Association of Directors of Medical Student Education in Psychiatry Annual Meeting; June 21, 2025; Boston, MA.

Ethical Approval
Write State University Institutional Review Board reviewed this project.

References

1. About intimate partner violence. U.S. Centers for Disease Control and Prevention. Updated February 11, 2026. Accessed May 22, 2026. https://www.cdc.gov/intimate-partner-violence/about/index.html
2. Peterson C, Kearns MC, McIntosh WL, et al. Lifetime economic burden of intimate partner violence among U.S. adults. *Am J Prev Med*. 2018;55(4):433-444. https://doi.org/10.1016/j.amepre.2018.04.049
3. Rodrigues M, Neaman A, Ditzer J, Talmon A. The impact of intimate partner violence on the mental and physical health of sexual and gender minorities: a comprehensive review of quantitative research. *Arch Sex Behav*. 2025;54(2):433-447. https://doi.org/10.1007/s10508-024-03023-z
4. Dokkedahl SB, Kirubakaran R, Bech-Hansen D, Kristensen TR, Elklit A. The psychological subtype of intimate partner violence and its effect on mental health: a systematic review with meta-analyses. *Syst Rev*. 2022;11(1):163. https://doi.org/10.1186/s13643-022-02025-z
5. Campbell JC, Lewandowski LA. Mental and physical health effects of intimate partner violence on women and children. *Psychiatr Clin North Am*. 1997;20(2):353-374. https://doi.org/10.1016/S0193-953X(05)70317-8
6. Sarkar NN. The impact of intimate partner violence on women's reproductive health and pregnancy outcome. *J Obstet Gynaecol*. 2008;28(3):266-271. https://doi.org/10.1080/01443610802042415
7. Yau JHY, Wong JYH, Fong DYT. Economic abuse as a form of intimate partner violence: a literature review of the instruments and mental well-being outcomes. *Violence Vict*. 2021;36(4): 479-492.
8. Kalra N, Hooker L, Reisenhofer S, Di Tanna GL, García-Moreno C. Training healthcare providers to respond to intimate partner violence against women. *Cochrane Database Syst Rev*. 2021; 5(5):CD012423. https://doi.org/10.1002/14651858.CD012423.pub2

Copyright © 2026 Petsinger et al. This is an open-access publication distributed under the terms of the Creative Commons Attribution license.

9. Renner LM, Wang Q, Logeais ME, Clark CJ. Health care providers' readiness to identify and respond to intimate partner violence. *J Interpers Violence.* 2021;36(19-20):9507-9534. https://doi.org/10.1177/0886260519867705

10. Barnard M, West-Strum D, Yang Y, Holmes E. Evaluation of a tool to measure pharmacists' readiness to manage intimate partner violence. *Pharmacy (Basel).* 2018;6(3):66. https://doi.org/10.3390/pharmacy6030066

11. Delungahawatta T, Dunne SS, Hyde S, et al. Advances in e-learning in undergraduate clinical medicine: a systematic review. *BMC Med Educ*. 2022;22(1):711. https://doi.org/10.1186/s12909-022-03773-1

12. Futures Without Violence CUES Videos. Health Partners on IPV + Exploitation. Accessed May 22, 2026. https://healthpartnersipve.org/resources/futures-without-violence-cues-videos/

13. Feltner C, Wallace I, Berkman N, et al. Appendix F Table 1: IPV screening instruments. In: *Screening for Intimate Partner Violence, Elder Abuse, and Abuse of Vulnerable Adults: An Evidence Review for the U.S. Preventive Services Task Force*. Agency for Healthcare Research and Quality; 2018. Accessed May 22, 2026. https://www.ncbi.nlm.nih.gov/books/NBK533715/table/appf.tab1/

14. Iorio-Morin C, Brisebois S, Becotte A, Mior F. Improving the pedagogical effectiveness of medical videos. *J Vis Commun Med*. 2017;40(3):96-100. https://doi.org/10.1080/17453054.2017.1366826

15. O'Neil HF, Perez RS, eds. Web-Based Learning: Theory, Research, and Practice. Routledge; 2013.

16. Short LM, Alpert E, Harris JM Jr, Surprenant ZJ. A tool for measuring physician readiness to manage intimate partner violence. *Am J Prev Med*. 2006;30(2):173-180. https://doi.org/10.1016/j.amepre.2005.10.009

17. Agresti A. *Categorical Data Analysis*. 2nd ed. Wiley; 2002. https://doi.org/10.1002/0471249688

18. R Core Team. R: A Language and Environment for Statistical Computing. R Foundation for Statistical Computing; 2023. https://www.R-project.org/

19. Christensen RHB. Ordinal: Regression Models for Ordinal Data. R package version 2023.12-4; 2023. https://CRAN.R-project.org/package=ordinal

Received: November 20, 2025
Accepted: June 1, 2026
Published: July 14, 2026

Copyright © 2026 Petsinger et al. This is an open-access publication distributed under the terms of the Creative Commons Attribution license.
